# Splenic B-cell lymphoma/leukemia with prominent nucleoli and spontaneous splenic rupture: a case report and literature review

**DOI:** 10.3389/fmed.2025.1693164

**Published:** 2026-01-07

**Authors:** Riwei Wang, Yun Xia, Haoru Liu

**Affiliations:** 1 Department of the Third General Surgery, Jiujiang City Key Laboratory of Cell Therapy, Jiujiang No.1 People’s Hospital, Jiujiang, China; 2 Department of Imaging, Jiujiang City Key Laboratory of Cell Therapy, Jiujiang No.1 People’s Hospital, Jiujiang, China; 3 Department of Pathology, Jiujiang City Key Laboratory of Cell Therapy, Jiujiang No.1 People’s Hospital, Jiujiang, China

**Keywords:** case report, emergency surgery, splenic B-cell lymphoma/leukemia with prominent nucleoli (SBLPN), splenomegaly, spontaneous splenic rupture

## Abstract

An 80-year-old man was admitted to our hospital for recurrent chest discomfort and shortness of breath over the past 2 years, with symptoms exacerbated by back pain over the past 3 days. The patient had a history of multiple comorbidities. Physical examination on admission revealed grade III splenomegaly. Peripheral blood tests revealed a white blood cell count of 27.03 × 10^9^/L, a lymphocyte percentage of 62.40%, and an absolute lymphocyte count of 16.87 × 10^9^/L. During hospitalization, the patient’s blood pressure suddenly dropped, and he developed shock. Computed tomography scan confirmed a rupture at the upper pole of the spleen, requiring emergency surgical exploration. Intraoperatively, the splenic rupture site was identified, and substantial intraperitoneal hemorrhage was noted. Therefore, total splenectomy was performed. The patient’s postoperative recovery was initially uneventful; however, chemotherapy was not administered due to poor physical status. Postoperative pathological examination, bone marrow smear, and flow cytometry analysis confirmed the diagnosis of splenic B-cell lymphoma/leukemia with prominent nucleoli (SBLPN). SBLPN is an extremely rare splenic B-lymphocyte neoplasm, with a small number of cases reported in the current literature.

## 1 Introduction

Spontaneous splenic rupture is a rare nontraumatic emergency in clinical practice that typically occurs in pathologically altered spleens. The common causes of splenic pathological changes are hematological disorders, inflammatory conditions, and neoplastic diseases (1). Splenic B-cell lymphoma/leukemia with prominent nucleoli (SBLPN) is a rare splenic B-lymphocyte neoplasm, accounting for 0.4% of all chronic lymphoproliferative disorders, with an annual incidence of 0.03 cases per 100,000 individuals (2). Its clinical manifestations usually include splenomegaly, lymphocytosis (without monocytopenia), and cytopenia-related symptoms (3). We herein report a case of SBLPN incidentally diagnosed after spontaneous splenic rupture, aiming to enhance the clinical understanding of this rare disease. That few cases have correlated splenic rupture pathology with the immunophenotypic spectrum of SBLPN. The key significance of this case is that it provides valuable insights into the diagnosis of SBLPN.

## 2 Case presentation

### 2.1 Clinical manifestations

On January 12, 2025, the patient was admitted with a chief complaint of “recurrent chest tightness and shortness of breath for 2 years, worsened by back pain over the past 3 days.” Two years prior, the patient experienced chest tightness and shortness of breath without obvious triggers. These symptoms were intermittent, lasting more than 10 min per episode, typically occurring after physical activity and resolving with rest, and were accompanied by bilateral lower extremity edema. Three days prior to admission, the patient experienced a recurrence of chest tightness and shortness of breath, accompanied by new-onset back pain. During hospitalization, he had a sudden hypotensive episode and exhibited signs of shock.

### 2.2 Physical examination on admission

Chest: Symmetrical thoracic contour, clear bilateral breath sounds.

Heart: Irregular cardiac rhythm (consistent with atrial fibrillation).

Abdomen: Splenomegaly extending beyond the umbilicus.

Extremities: Bilateral lower extremity edema.

### 2.3 Medical history

The patient had a long-standing history of atrial fibrillation and was on long-term oral rivaroxaban therapy. Furthermore, he had a history of chronic obstructive pulmonary disease (COPD) and was receiving long-term tiotropium bromide inhalation treatment.

### 2.4 Auxiliary examinations

**Figure 1:**
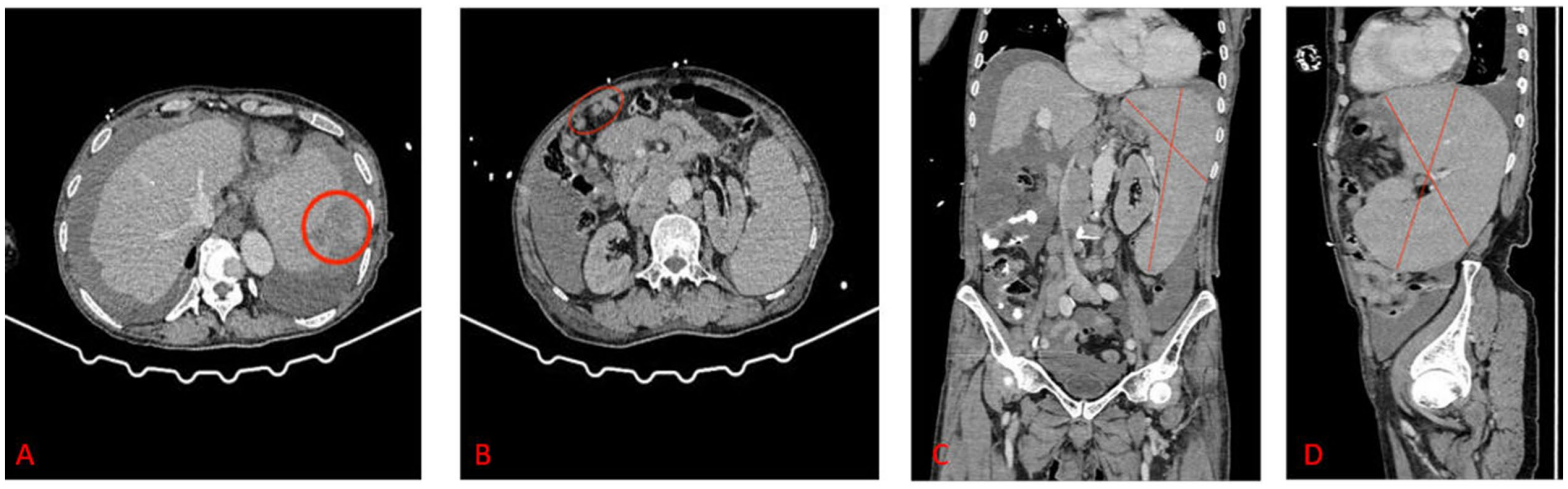
CT scan indicated a rupture at the superior pole of the spleen, hemoperitoneum, and multiple enlarged lymph nodes. **(A)** The red circle indicated the rupture point. **(B)** The red oval area indicated multiple enlarged lymph nodes. **(C)** Massive splenomegaly in the coronal plane (the red-line marked area). **(D)** Massive splenomegaly in the sagittal plane (the red-line marked area).

Imaging: Abdominal computed tomography scan revealed a rupture at the upper pole of the spleen, hemoperitoneum, and multiple enlarged lymph nodes (Figure 1).

Peripheral Blood Tests: White blood cell count, 27.03 × 10^9^/L (normal range: 4–10 × 10^9^/L); red blood cell count, 3.81 × 10^12^/L; hemoglobin, 111 g/L; platelet count, 85 × 10^9^/L; lymphocyte percentage, 62.40%; absolute lymphocyte count, 16.87 × 10^9^/L; creatinine, 429 μmol/L; uric acid, 601 μmol/L; N-terminal pro-B-type natriuretic peptide, 14,449.00 pg./mL.

### 2.5 Preliminary diagnosis

Spontaneous splenic rupture, hemorrhagic shock, acute exacerbation of chronic cardiac insufficiency (New York Heart Association class III), acute exacerbation of COPD, atrial fibrillation, rapidly progressive glomerulonephritis.

### 2.6 Surgical outcome

On January 16, 2025, emergency surgery was performed. Intraoperative exploration revealed approximately 1,500 mL of bloody fluid in the abdominal cavity, a cirrhotic liver, and an enlarged spleen measuring approximately 20 × 15 cm. A laceration (6-cm long) was observed on the dorsal surface of the splenic upper pole. Total splenectomy was performed, and the surgery proceeded without complications.

### 2.7 Postoperative recovery

Immediately after surgery, the patient was transferred to the intensive care unit for close monitoring and intensive care. A combination of norepinephrine and dopamine was administered to stabilize blood pressure, and blood transfusion was performed to address massive hemorrhage. Postoperative re-evaluation of peripheral blood tests revealed a white blood cell count of 56.87 × 10^9^/L, lymphocyte count of 31.26 × 10^9^/L, and creatinine of 668 μmol/L. Owing to the presence of concurrent pulmonary infection, anti-infective therapy with cefoperazone–sulbactam in combination with moxifloxacin was initiated. Due to persistent elevation in creatinine levels, continuous renal replacement therapy (CRRT) was implemented.

Once the patient’s condition stabilized, he was transferred to a general ward, where he received level-I nursing care and continued antibiotic therapy. Nutritional support was administered via enteral and parenteral routes. After a period of treatment, the patient regained the ability to pass flatus and stool. However, he failed to regain his pre-admission mental and physical status. Due to intolerance to CRRT, he opted for voluntary discharge against medical advice.

### 2.8 Postoperative diagnostic results

**Figure 2:**
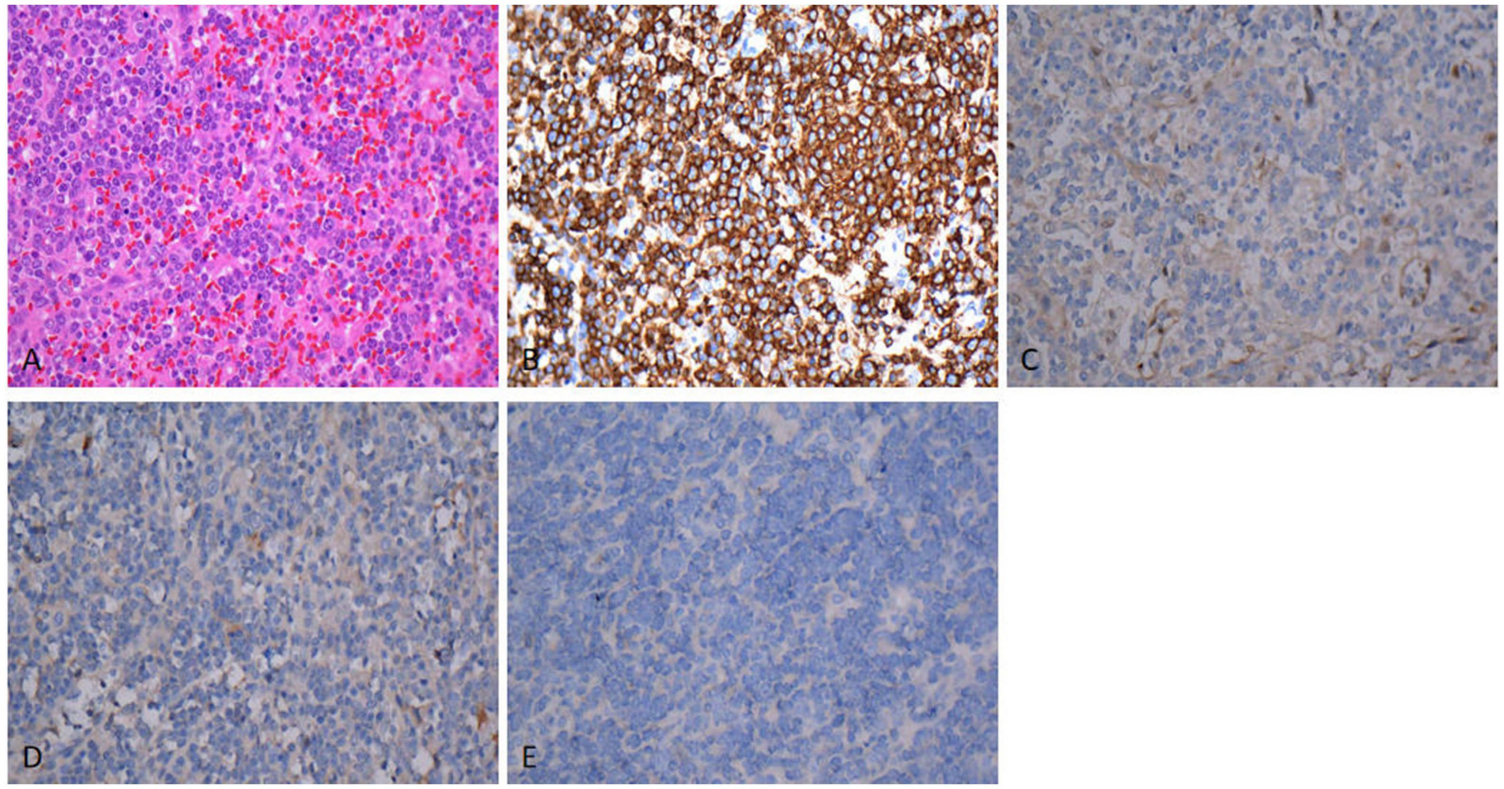
**(A)** Hematoxylin–eosin staining showed that tumor cells diffusely scattered, exhibiting infiltrative growth. The cells were moderate size with prominent nucleoli. Immunohistochemistry revealed positive expression of CD20 **(B)** and negative expression of Cyclin D1 **(C)**, CD123 **(D)**, BRAF **(E)**. Magnification: AX400, BX400, CX400, DX400, EX400.

Pathological and Immunohistochemical Examinations: As depicted in Figure 2, routine hematoxylin–eosin staining demonstrated diffuse infiltration and growth of medium-sized tumor cells with prominent nucleoli. Immunohistochemistry revealed positive expression of CD20 (Figure 2B) and negative expressions of Cyclin D1 (Figure 2C), CD123 (Figure 2D), and BRAF (Figure 2E) (magnification: ×400 for all panels). Splenectomy histology confirmed that the rupture resulted from infiltration rather than infarction.

**Figure 3:**
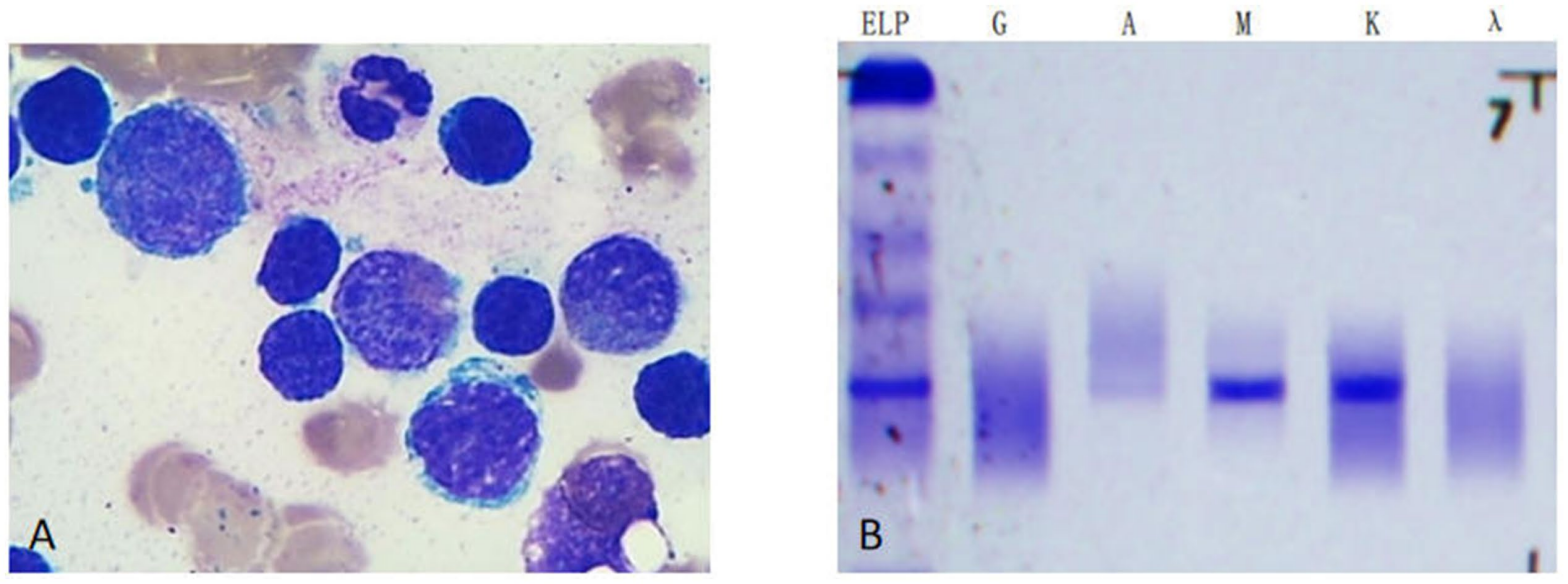
**(A)** The bone marrow aspiration revealed 53.2% lymphocytes, with lymphoma-like cells accounted for 50%. Such cells exhibited smaller cytoplasmic body, regular nuclear morphology, condensed chromatin, and nucleoli in some case, with scant cytoplasm. (Wright-Giemsa stain, X1000). **(B)** Serum immunofixation electrophoresis indicated a monoclonal immunoglobulin type of IgM-K.

Bone Marrow Smear: As illustrated in Figure 3A, bone marrow aspiration revealed 53.2% lymphocytes, 50% of which were lymphoma-like cells. These cells exhibited a small cytoplasmic volume, regular nuclear morphology, condensed chromatin, occasional nucleoli, and scanty cytoplasm (Wright-Giemsa stain, ×1,000). Serum immunofixation electrophoresis detected a monoclonal IgM-*κ* immunoglobulin (Figure 3B).

**Figure 4:**
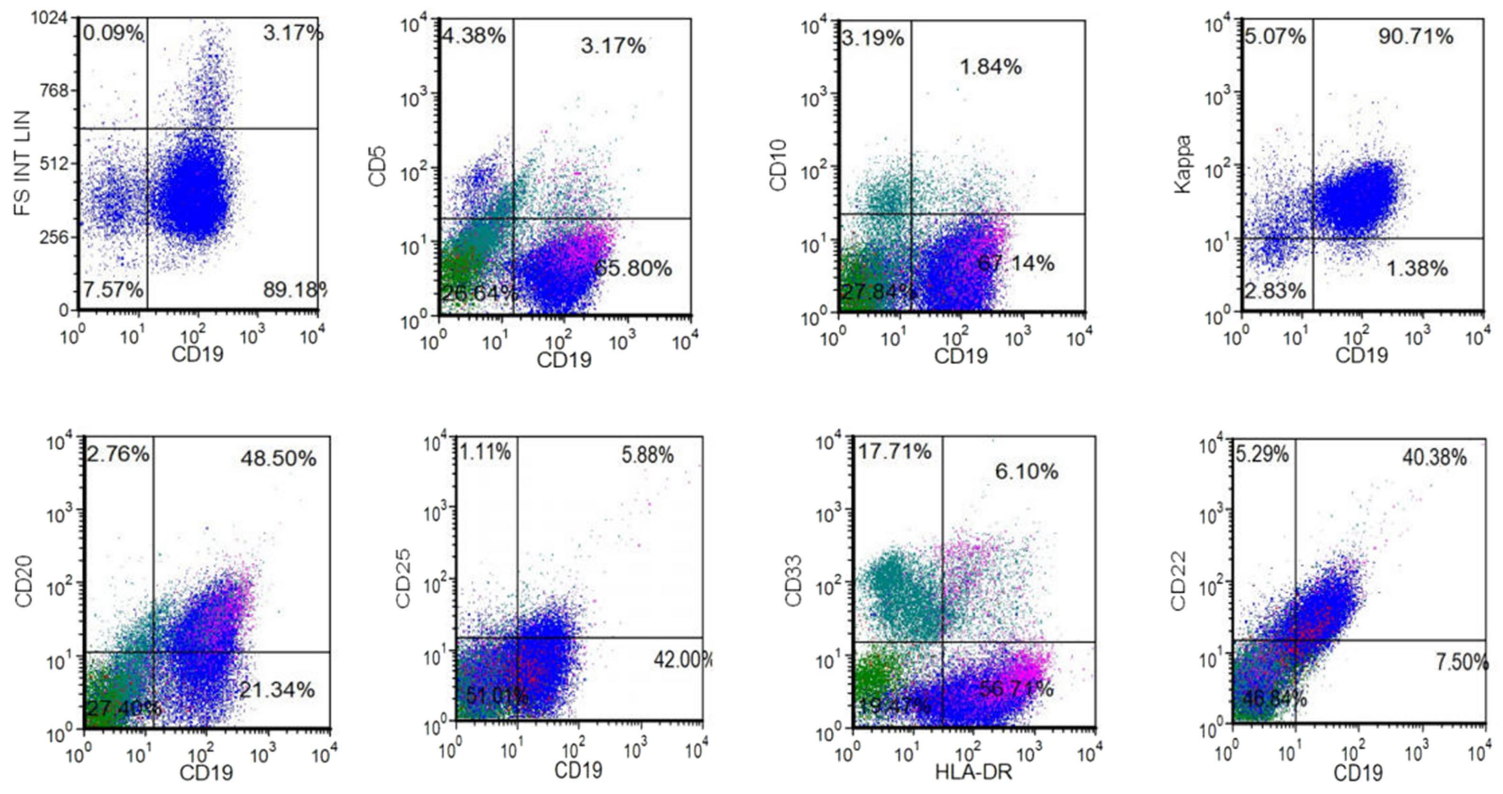
The results of flow cytometry indicated the presence of approximately 67.14% mature B lymphocytes in the submitted specimen. The immunophenotype findings showed expression of Kappa, CD19, CD22, HLA-DR and CD20 dim, and negative expression of CD10, CD5, CD25, CD33. The flow cytometry results were consistent with CD5-negative, CD10-negative mature B-cell lymphoma/leukemia.

Flow Cytometry: As shown in Figure 4, flow cytometry analysis revealed approximately 67.14% mature B lymphocytes in the specimen. The immunophenotype was characterized by positive Kappa, CD19, CD22, HLA-DR, and dim CD20 expressions and negative CD10, CD5, CD25, and CD33 expressions, consistent with CD5^−^CD10^−^ mature B-cell lymphoma/leukemia.

**Figure 5:**
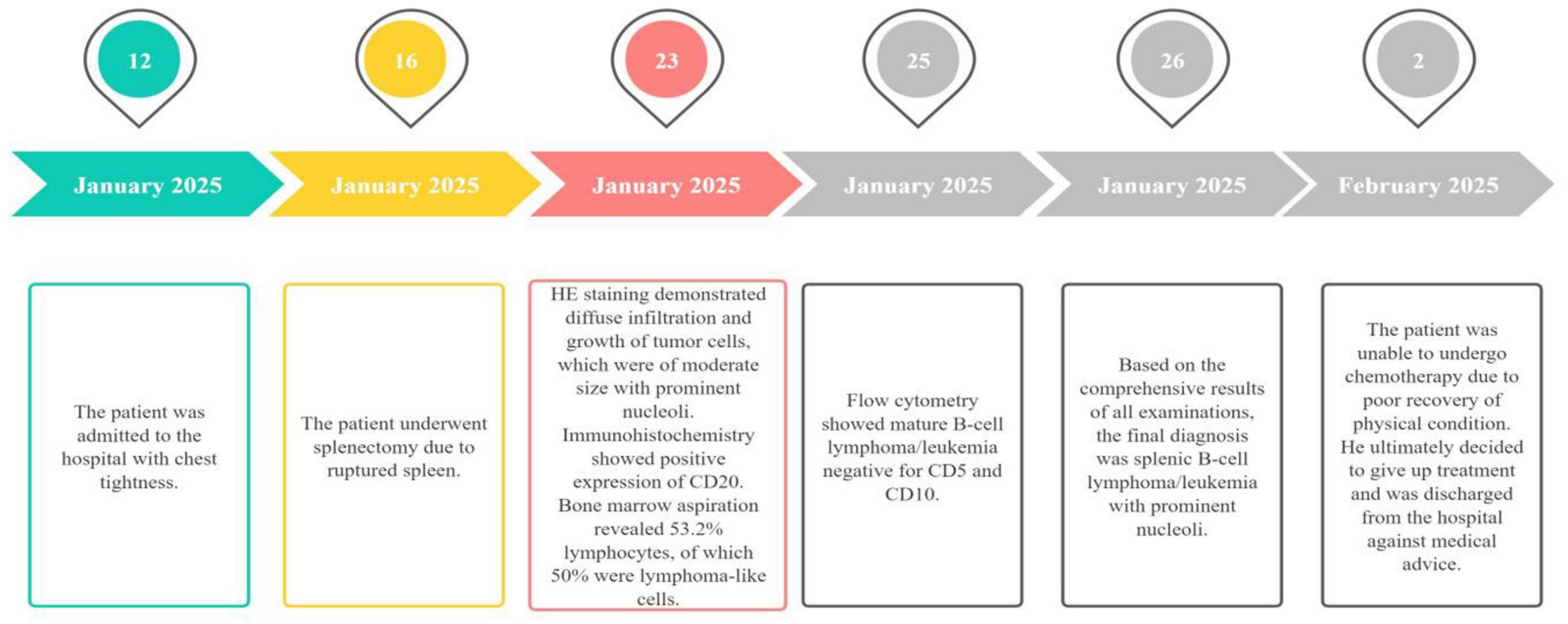
The timeline of disease.

A comprehensive analysis of the above findings confirmed the final diagnosis of SBLPN. Figure 5 presents the timeline of disease diagnosis for the patient.

## 3 Discussion

Splenic lymphomas constitute a heterogeneous group of lymphoid neoplasms with overlapping clinical features and ambiguous laboratory findings. To standardize the classification of such cases, the World Health Organization Classification of Haematolymphoid Tumours, 5th edition, has officially introduced the term “SBLPN” (4). Previously referred to as “hairy-cell variant,” this nomenclature highlights the biological distinction between SBLPN and hairy-cell leukemia (HCL), despite morphological similarities between their leukemic cells (5).

SBLPN encompasses several previously categorized splenic lymphomas, including hairy-cell leukemia variant (HCL-V), B-prolymphocytic leukemia (B-PLL), and subsets of splenic marginal zone lymphoma (SMZL) and splenic diffuse red pulp small B-cell lymphoma (SDRPL), with histological progression. Distinguishing these neoplasms in clinical practice is challenging owing to their variable clinical courses and divergent therapeutic requirements. Establishing a definitive diagnosis, particularly in complex cases, is crucial for integrating clinical data, morphological features, histological findings, immunophenotypic profiles, and molecular characteristics (6).

SBLPN is characterized by the cytomorphology of medium-to-large atypical lymphoid cells with a single prominent large nucleolus, resembling prolymphocytes, and indistinct cytoplasmic projections, similar to HCL cells. As a newly defined entity, SBLPN lacks dedicated clinical series, and its molecular features are mainly extrapolated from existing HCL-V and B-PLL studies (4).

HCL-V is a rare subtype of mature B-cell chronic lymphoproliferative disorder (B-CLPD) (7). It has similar features as other splenic B-cell lymphomas: like HCL, HCL-V cells have cytoplasmic protrusions; unlike HCL, they typically have prominent nucleoli (8). Immunophenotypically, HCL-V cells are positive for CD19, CD20, CD22, and IgD but negative for Cyclin D1, Annexin A1, and CD25; the expressions of CD103 and CD123 are usually weak or absent (4, 9). In the present case, SBLPN cells exhibited positive Kappa, CD19, CD22, HLA-DR, and dim CD20 expressions and negative CD10, CD5, CD25, and CD33 expressions. Serum immunofixation electrophoresis revealed a monoclonal IgM-*κ* immunoglobulin—findings that are slightly inconsistent with previous reports on HCL-V, potentially reflecting the phenotypic heterogeneity of SBLPN.

Genetically, HCL-V typically lacks mutations in BRAF p. V600E, KLF2, and CDKN1B, whereas MAP2K1 mutations are relatively common (occurring in 38–42% of cases) (10). In this case, next-generation sequencing did not detect BRAF V600E mutation, consistent with the genetic profile of HCL-V reported in the literature. SBLPN is pathologically characterized by significant splenic red pulp enlargement caused by diffuse infiltration of leukemic cells, accompanied by marked sinusoidal involvement (11). The most common initial manifestation is splenomegaly, occurring in 85% of cases, whereas hepatomegaly is present in fewer than one-third of cases; peripheral lymphadenopathy and involvement of other organs are relatively rare (9).

B-PLL was initially described as an aggressive lymphoma, marked by massive splenomegaly, minimal or absent lymphadenopathy, substantial peripheral blood lymphocytosis (with prolymphocytes accounting for >55% of leukocytes), and bone marrow involvement (4). Morphologically, B-PLL cells are medium-sized with a central nucleolus, indistinct cytoplasmic borders, and variable cytoplasmic processes. Immunophenotypically, B-PLL significantly overlaps with other B-CLPDs, demonstrating strong expression of surface membrane immunoglobulin, CD19, CD20, CD22, CD79a, and FMC7, with absent or weak CD23 and CD5 expressions (12). Owing to the overlap in clinical manifestations between B-PLL and other B-CLPDs, conventional B-CLPD-targeted therapies yield only partial efficacy in patients with B-PLL, who often experience early recurrence (13).

SBLPN mainly affects elderly individuals, particularly men. The identification of SBLPN in the context of spontaneous splenic rupture highlights the need to consider occult lymphoproliferative disorders in elderly patients with atraumatic splenic bleeding. The immunophenotypic findings in our case report showed positive Kappa, CD19, CD22, HLA-DR, and CD20 dim expressions and negative CD10, CD5, CD25, and CD33 expressions. The flow cytometry results were consistent with CD5-negative, CD10-negative mature B-cell lymphoma/leukemia. It differs to some extent from previously reported splenic lymphoma. According to current literature, differentiation of SBLPN from HCL, SMZL, SDRPL, and SMZL (splenic marginal zone lymphoma) is crucial. Their immunophenotypes exhibit overlap and distinctions (14), as presented in Table 1. It is associated with poor treatment response and a guarded prognosis: therapies effective for HCL-V demonstrate limited efficacy in SBLPN, and there are no standardized management guidelines. Splenectomy is considered to be a beneficial intervention, as it may enhance the efficacy of subsequent chemotherapy (11). The chemotherapy regimen of rituximab + purine nucleoside analogs demonstrates superior efficacy to monotherapy (15). The results of combination therapy reported in previously published small-series studies indicated that treatment with rituximab combined with cladribine has achieved favorable outcomes (16). Ibrutinib is an oral small-molecule Bruton’s tyrosine kinase inhibitor that has been approved for the treatment of various B-cell lymphoproliferative disorders (17). It exerts certain inhibitory effects on SBLPN. As half of patients with SBLPN harbor activating MAP2K mutations, MEK inhibitors (trametinib) have also exhibited significant therapeutic potential. At present, clinical evaluations of the MEK inhibitor binimetinib for treatment-refractory HCL-c and SBLPN are ongoing (18). Splenectomy is associated with postoperative complications and infection risks, which are more pronounced in the elderly. Therefore, rituximab monotherapy is often recommended and well tolerated in such patients (14).

**Table 1:** Differential immunophenotype of SBLPN, SDRPL, SMZL, and HCL.

Immunophenotype	SBLPN	SDRPL	SMZL	HCL
CD19	Positive	Positive	Positive	Positive
CD22	Positive	ND	Positive	Positive
CD20	Dim positive	Positive	Positive	Positive
CD10	Negative	Negative	Negative	Negative
CD5	Negative	Negative	Negative	Negative
CD25	Negative	Negative	ND	positive
CD33	Negative	ND	ND	ND
CD23	ND	Negative	Negative	Negative
CD11c	Positive	Negative	ND	positive
CD103	Positive	Negative	Negative	positive
CD123	Negative	Negative	Negative	positive
Kappa	Positive	ND	ND	ND
HLA-DR	Positive	ND	ND	ND
IgM-κ	Positive	ND	ND	ND
FMC7	ND	ND	Positive	
Annexin A1	ND	Negative	Negative	positive
Cyclin D1	Negative	Negative	Negative	ND

A previous study on chronic myeloid leukemia described spontaneous splenic rupture resulting from leukemic infiltration, highlighting shared mechanisms of splenic vulnerability across hematologic malignancies (19). The majority of splenic ruptures caused by lymphoma have been reported as case studies. The possible mechanisms include (1) tumor cell infiltration of the spleen leading to splenomegaly, which compresses blood vessels and causes splenic infarction, resulting in hemorrhage; (2) lymphomatous infiltration of the bone marrow suppressing megakaryocyte proliferation, resulting in thrombocytopenia and potentially bleeding; and (3) lymphoma-induced enlargement causing disruption of the splenic capsule and subsequent hemorrhage.

Re-examining the patients’ diagnosis and treatment, he was admitted to the hospital due to chest tightness and discomfort. Physical and imaging examinations revealed splenomegaly. While awaiting a definitive diagnosis of the disease, the patient experienced spontaneous splenic rupture and was given surgical treatment. The final diagnosis of SBLPN was ultimately confirmed via splenic pathological examination and bone marrow analysis. Unfortunately, the patient did not receive further chemotherapy, preventing follow-up on treatment outcomes for SBLPN.

Splenectomy for the definitive diagnosis of splenic lymphoma is often impractical in actual clinical practice. The final diagnosis of SBLPN relies on bone marrow cytology, imaging studies, and peripheral blood smear examination.

## 4 Conclusion

The present case underscores the complexity of managing elderly patients with multiple comorbidities who present with spontaneous splenic rupture. In this case, emergency splenectomy not only addressed the life-threatening hemorrhage but also provided critical tissue samples for establishing the definitive diagnosis of SBLPN, thereby laying the groundwork for potential subsequent treatment. Owing to the rarity of SBLPN, more case reports and multicenter studies are warranted to further elucidate its clinical features, molecular mechanisms, and optimal therapeutic strategies. As anticoagulant therapy (rivaroxaban) can worsen hemorrhagic outcomes in splenic malignancy, early imaging and a multidisciplinary evaluation are recommended for elderly patients with splenomegaly and hemodynamic instability.

## Data Availability

The raw data supporting the conclusions of this article will be made available by the authors, without undue reservation.
